# Qishen granules inhibit myocardial inflammation injury through regulating arachidonic acid metabolism

**DOI:** 10.1038/srep36949

**Published:** 2016-11-11

**Authors:** Chun Li, Jing Wang, Qiyan Wang, Yi Zhang, Na Zhang, Linghui Lu, Yan Wu, Qian Zhang, Wei Wang, Yong Wang, Pengfei Tu

**Affiliations:** 1 Modern Research Center for Traditional Chinese Medicine, Beijing University of Chinese Medicine, Beijing 100029, China; 2 Basic Medical College, Beijing University of Chinese Medicine, Beijing 100029, China; 3 School of Chinese Materia Medica, Beijing University of Chinese Medicine, Beijing 100102, China; 4 Center of Scientific Experiment, Beijing University of Chinese Medicine, Beijing 100029, China

## Abstract

Qishen granules (QSG), a traditional Chinese medicine, have been prescribed widely in the treatment of coronary heart diseases. Previous studies demonstrated that QSG had anti-inflammatory and cardio-protective effects in mice with acute myocardial infarction (AMI). However, the mechanisms by which QSG attenuate inflammation and prevent post-AMI heart failure (HF) are still unclear. In this study, we explored the anti-inflammatory mechanisms of QSG by *in vitro* and *in vivo* experiments. A novel inflammatory injury model of H9C2 cells was induced by lipopolysaccharide (LPS)-stimulated macrophage-conditioned media (CM). An animal model of AMI was conducted by ligation of left anterior descending (LAD) coronary artery in mice. We found that QSG inhibited release of cytokines from LPS-stimulated RAW 264.7 macrophages and protected H9C2 cardiac cells against CM-induced injury. *In vivo* results showed that QSG administration could improve cardiac functions and alter pathological changes in model of AMI. QSG regulated multiple key molecules, including phospholipases A2 (PLA2), cyclooxygenases (COXs) and lipoxygenases (LOXs), in arachidonic acid metabolism pathway. Interestingly, QSG also targeted TNF-α-NF-κB and IL-6-JAK2-STAT3 signaling pathways. Taken together, QSG achieve synergistic effects in mitigating post-AMI HF by regulating multiple targets in inflammatory pathways. This study provides insights into anti-inflammatory therapeutics in managing HF after AMI.

Although rapid progress has been made in the treatment of acute myocardial infarction (AMI), mortality rate caused by AMI is still high and patients surviving AMI are at a high risk of developing heart failure (HF), indicating that current therapies still miss one or more critical pathological mechanisms^1^,^2^. Therefore, investigation into the mechanisms of AMI is important in developing new strategies to prevent HF after AMI.

Ischemic injury initiates an intense inflammatory response that leads to further dysfunction and HF^3^. In this process, the macrophages play an important role in aggravating inflammation and promoting cardiac fibrosis and apoptosis^4^. The healthy myocardium hosts a considerable number of macrophages, which are among the largest cardiac resident cell populations, trailing only fibroblasts, myocytes, and endothelial cells^4^,^5^. When cardiac tissues suffer ischemic injuries, local macrophages are activated and release pro-inflammatory cytokines. Soon after AMI, ischemic tissue attracts abundant inflammatory monocytes which are recruited to ischemic site and then differentiated into inflammatory macrophages. Both local and recruited macrophages generate abundant inflammatory cytokines, cathepsins and matrix metalloproteinases in order to prepare for tissue repairing and rebuilding, which however often lead to cardiac damage^6^.

Several signaling pathways, including PLA2-COXs/LOXs and IL-6-JAK2-STAT3, are involved in the inflammatory process. In PLA2-COXs/LOXs pathway, arachidonic acids (AA) are hydrolyzed from membrane phospholipids by catalysis of phospholipases A2 (PLA2). AA can be further metabolized by cyclooxygenases (COXs) and lipoxygenases (LOXs) into biologically active eicosanoid products such as prostaglandins (PGs), hydroxyeicosatetraenoic acids (HETEs) and leukotrienes (LTs)^7^. Elevated eicosanoid production contributes to maladaptive changes such as inflammation and fibrosis^8^. In addition, interleukin-6 (IL-6) cytokine binds with glycoprotein-130 (gp130) and activates Janus kinase2/signal transducer and activator of transcription 3(JAK2/STAT3)^9^,^10^. STAT3 activation promotes inflammation, adverse ventricular remodeling and heart failure^11^. Targeting the key molecules in these pathways might be the mechanisms by which drugs exert anti-inflammatory effect.

Traditional Chinese medicine (TCM) has been applied in the treatment of AMI and prevention of HF for thousands of years, and an increasing number of herbal formulae have been proven to be effective^12^. Qishen granules (QSG) are prepared from a composition of six herbs of TCM, including two star herbs, *Radix Astragali Mongolici* (‘huang-qi’ in Chinese) and *Salvia Miltiorrhizabunge* (‘dan-shen’ in Chinese), and four other adjunctive herbs: *Flos Lonicerae, Scrophularia, Radix Aconiti Lateralis Preparata,* and *Radix Glycyrrhizae*. This formula is widely manufactured in China in accordance with the China Pharmacopoeia standard of quality control. The fingerprint of QSG was analyzed by HPLC-IT-TOF-MS and the typical chromatograms were shown in Supplement Data 1. It has been reported that QSG could inhibit inflammation and prevent post-AMI remodeling^13^. However, the underlying mechanisms by which QSG attenuate inflammation in macrophages and myocardial cells of cardiac tissue remain unclear.

In this study, we explored the effects of QSG on macrophages activated by lipopolysaccharide (LPS). We also established a novel H9C2 cardiac cell injury model induced by conditioned-media (CM) from LPS-stimulated macrophages and investigated the protective effects of QSG on H9C2 cardiac cells. The anti-inflammatory mechanism of QSG was further investigated in an animal model of AMI induced by ligation of left anterior descending (LAD) coronary artery in mice. Our extensive *in vivo* and *in vitro* studies will reveal the potential targets of QSG in inflammatory pathways and provide potential therapeutic approaches in the management of heart failure.

## Results

### QSG inhibited the production of inflammatory mediators in LPS-induced RAW264.7 cells by suppressing NF-κB and inhibiting the expression of COX2

**Figure 1:**
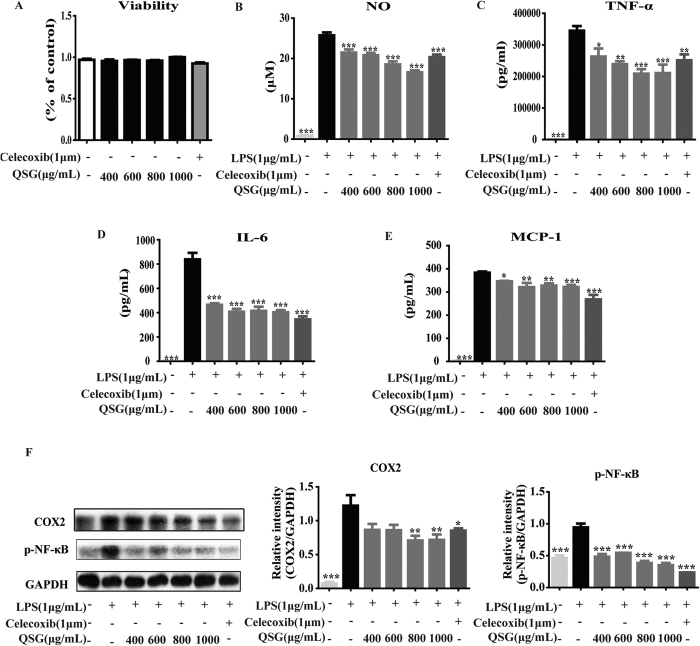
QSG inhibited the production of inflammatory mediators in LPS-stimulated RAW264.7 cells by inactivating p-NF-κB pathway and inhibiting expression of COX2. (**A**) RAW 264.7 cells were treated with QSG (400, 600, 800 or 1000 μg/mL) and Celecoxib (1 μM) for 24 h, and the cell viability was detected by CCK-8 assay. QSG and Celecoxib were shown to have no cytotoxicity in RAW 264.7 cells. (**B**–**E**) RAW 264.7 cells were treated with LPS (1 μg/mL) in the absence or presence of QSG (400, 600, 800 or 1000 μg/mL) for 24 h and the releases of NO, TNF-α, IL-6 and MCP-1 in cell supernatants were detected by Griess and ELISA assay. (**F**) The protein expressions of COX2 and p-NF-κB were detected by western blot analysis. All data were presented as means ± SEM from independent experiments performed in triplicate. Comparisons were made between LPS-treated group and each of the other groups. *P < 0.05, **P < 0.01, ***P < 0.001.

In our previous study, we demonstrated that QSG exerted anti-inflammatory effects through inhibiting release of TNF-α and IL-6^13^. In this study, we confirmed that QSG could suppress LPS-induced inflammation in RAW264.7, a mouse macrophage-like cell line. Treatment of cells with 400–1000 μg/mL QSG and positive control drug Celecoxib (1 μM) showed no cytotoxicity in RAW 264.7 (Fig. 1A). QSG significantly decreased LPS-induced release of NO, TNF-α, IL-6 and MCP-1 (Fig. 1B–E). Western blot analysis also showed that QSG effectively inhibited expressions of some pivotal inflammatory proteins such as COX2 and phosphorylated NF-κB (Fig. 1F). Taken together, these results suggested that QSG could inhibit LPS-induced activation of macrophages and had potent anti-inflammatory effect by suppressing expressions of COX2 and p-NF-κB.

### Inflammatory model of cardiac H9C2 cells was induced by LPS-stimulated macrophage-conditioned media

**Figure 2:**
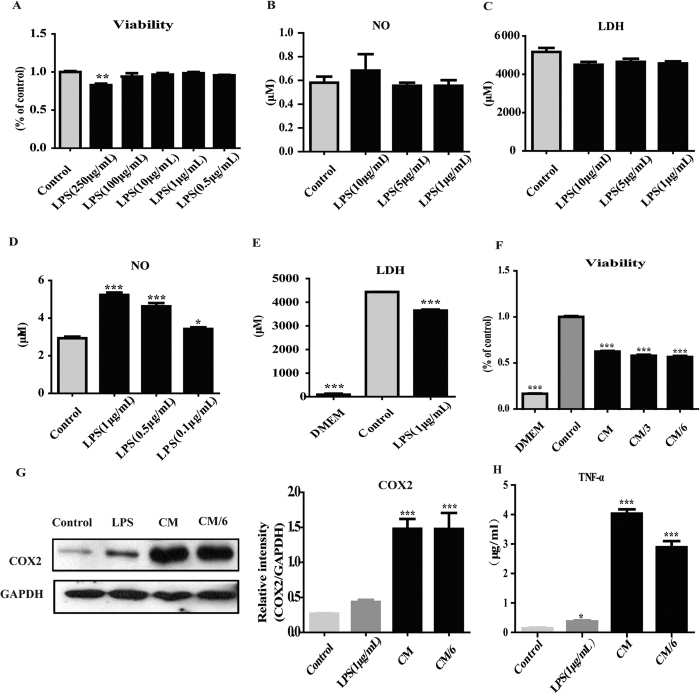
Inflammatory model of cardiac H9C2 cells was established by LPS-stimulated macrophage-conditioned media. (**A**) H9C2 cells were treated with LPS (0.5–250 μg/mL) for 24 h, and the cell viability was then detected by CCK-8 assay. Cell viabilities were similar between LPS-treated H9C2 cells and DMEM-treated cells. (**B**–**C**) The releases of NO and LDH were detected by Griess and LDH Cytotoxicity Assay. Productions of NO and LDH showed no significant difference among cells with different treatments. (**D**) RAW 264.7 cells were treated with LPS (1, 0.5, 0.1 μg/mL) for 24 h and NO productions were detected by Griess assay. LPS at the concentration of 1 μg/mL could increase the production of NO in RAW264.7 cells by 100%, compared with that in DMEM-treated control group, and this concentration was used for further studies. (**E**) LDH concentrations in supernatants collected from DMEM (containing 10% FBS)-treated macrophage cells and LPS-treated macrophage cells were much higher than that in FBS-free DMEM media. (**F**) H9C2 cells were incubated with three different concentrations of CM for 24 h, and the cell viability was detected by CCK8 assay. Cell viabilities were reduced by approximately 40% in CM (three concentrations)-stimulated H9C2 cells, compared with that in DMEM-treated cells (CM: conditioned media. CM/3: CM were diluted to one third of its original concentration. CM/6: CM were diluted to one six of its original concentration). (**G**) The protein expression of COX2 was detected by western blot analysis. CM increased expression of COX2 and TNF-α in H9C2 cells. (**H**) The protein expression of TNF-α was detected by Elisa analysis. CM increased expression of TNF-α in H9C2 cells. All data are presented as means ± SEM from independent experiments performed in triplicate. *P < 0.05, **P < 0.01, ***< 0.001. Comparisons were made between DMEM-treated cells in control group and each of the other groups.

After validating that QSG had anti-inflammatory effect on LPS-stimulated macrophages, we further explored the effects of QSG on cardiac H9C2 cells. An inflammatory cardiac cell model had to be established first. It was shown that 1–10 μg/mL LPS had no cytotoxic effect on H9C2 cardiac cells (Fig. 2A). Moreover, 1–10 μg/mL LPS had no effects on the production of NO and LDH in H9C2 cells (Fig. 2B,C), indicating that LPS isn’t an ideal agent for inducing inflammation in H9C2 cells. As cytokines released from macrophages play an important role in the progression of inflammation in ischemic heart tissue, we tried to induce an inflammatory cell model that simulates the environment *in vivo*. We successfully established a novel inflammatory H9C2 model by incubating cells with macrophage-conditioned media (CM). LPS at the concentration of 0.1–1 μg/mL could stimulate production of NO in RAW 264.7 cells and 1 μg/mL was chosen as the LPS dosage to activate RAW264.7 macrophage cells, as this dosage of LPS could induce twice the amount of NO released by macrophage cells as that was released in control DMEM-treated cells (Fig. 2D). Supernatants collected from LPS (1 μg/mL) –stimulated RAW264.7 cells were used as CM to incubate H9C2 cells for 24 h to induce inflammation. It was detected that LDH in supernatant collected from DMEM-treated macrophage cells was very high compared with that in FBS-free DMEM media (Fig. 2E). LDH in supernatant collected from LPS-treated cells was also significantly higher than that in FBS-free DMEM media, suggesting that FBS contains high concentrations of LDH (Fig. 2E). To exclude interference from FBS, H9C2 cells were cultured in FBS-free DMEM in the following experiments. CM were diluted to one third (CM/3) and one sixth (CM/6) of its original concentration by DMEM and the diluted CM were also applied to incubate H9C2 cells. Cell viability assay showed that CM, at three different concentrations, reduced H9C2 cell viability by approximately 40% (Fig. 2F). Moreover, expressions of COX2 in CM-stimulated H9C2 cells were much higher than those in LPS-stimulated cells or control DMEM-incubated cells (Fig. 2G). Furthermore, TNF-α, an inflammatory marker, was also detected. Level of TNF-α in LPS-stimulated H9C2 increased. Expressions of TNF-α in CM-stimulated H9C2 cells were much higher than those in LPS-stimulated cells or control DMEM-incubated cells (Fig. 2H). These results indicate that the *in vitro* inflammatory cardiac cell model was successfully established by induction of CM.

### QSG inhibited CM-induced inflammation in H9C2 cells by inactivating NF-κB pathway and suppressing the expression of COX2

**Figure 3:**
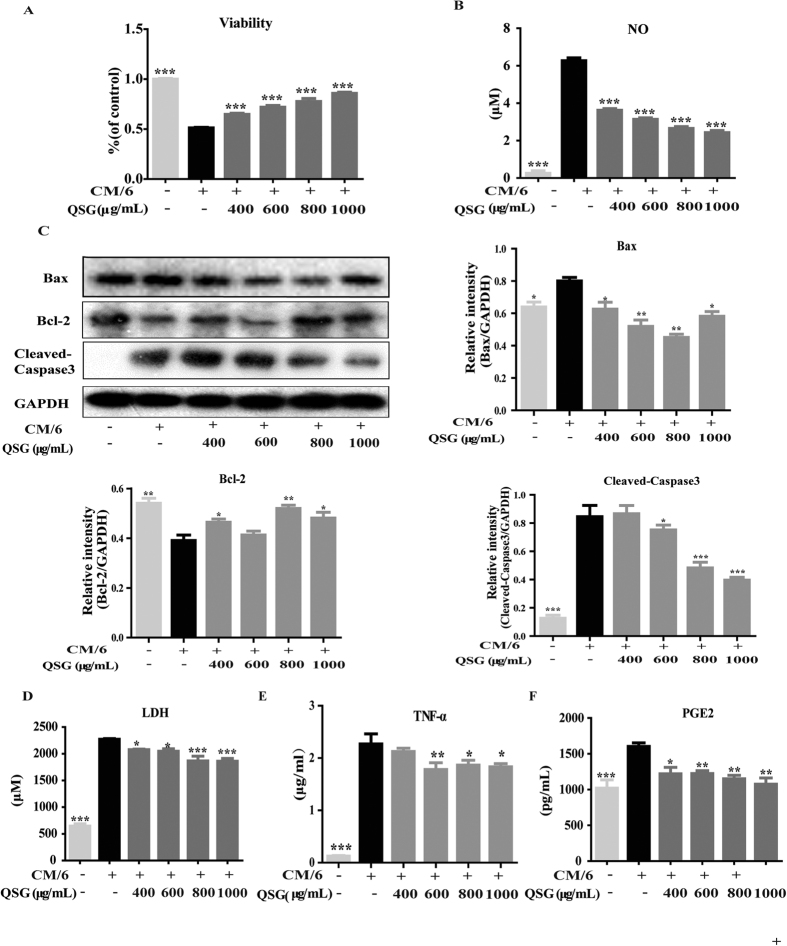
QSG protected cardiac H9C2 cells against inflammation induced by macrophage-conditioned media. (**A**) H9C2 cells were treated with LPS (1 μg/mL)-induced CM and QSG (0, 400, 600, 800 or 1000 μg/mL) for 24 h. The cell viability was detected by CCK8 assay. QSG increased cell survival rate. (**B**) The releases of NO in H9C2 cell supernatants was detected by Griess and ELISA assay. CM increased release of NO and pretreatment with QSG (400, 600, 800 or 1000 μg/mL) inhibited the production of the cytokines. (**C**) H9C2 cells were treated with CM/6 and QSG. Expressions of Bax, Bcl-2 and Caspase3 were detected by western blotting. CM/6 increased expressions of Bax and Caspase3 and reduced expression of Bcl-2. Pretreatment with QSG (400, 600, 800 or 1000 μg/mL) reversed CM/6 induced changes of these proteins. (**D**–**F**) The releases of LDH, TNF-α and PGE2 in H9C2 cell supernatants were detected by Griess and ELISA assay. CM increased release of these mediators and pretreatment with QSG (400, 600, 800 or 1000 μg/mL) inhibited the production of the cytokines. All data are presented as means ± SEM from independent experiments performed in triplicate. Comparisons were made between CM-treated group and each of the other groups. *P < 0.05, **P < 0.01, ***P < 0.001.

After establishing CM-induced inflammatory H9C2 cell model, we further explored the anti-inflammatory effects of QSG on cardiac cells. CM induced cellular apoptosis in H9C2 cells and QSG increased cell viability (Fig. 3A). As Bax, Bcl-2 and Caspase-3 (Cas-3) are the vital proteins in the apoptotic signaling pathways^14^,^15^, we detected their expression levels in CM-induced H9C2 cells with or without QSG treatment. The results showed that CM dramatically increased expressions of Bax and Caspase compared with the control group, whereas pretreatment of cells with QSG at the concentration of 600–1000 μg/mL inhibited CM-induced expression of Bax and Caspase-3 (Fig. 3C). Level of Bcl-2 in model group was much lower than control group and QSG up-regulated expression of Bcl-2 (Fig. 3C). CM also induced release of NO, LDH, TNF-α, and PGE2 in H9C2 cells and pretreatment of H9C2 cells with QSG attenuated release of these inflammatory mediators (Fig. 3B,D–F).

**Figure 4:**
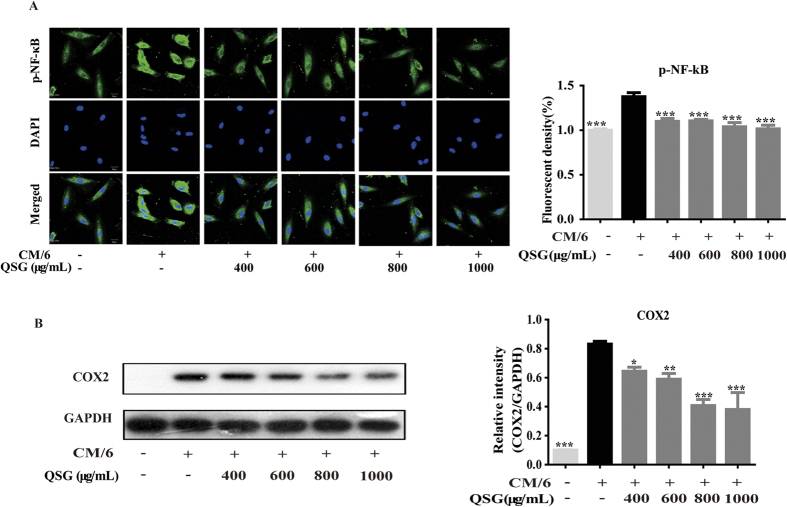
QSG inhibited expressions of p-NF-κB and COX2 in CM-stimulated H9C2 cells. (**A**) The protein expressions of p-NF-κB were detected by immunofluorescence assay and analyzed by the software of Image J. QSG in different dosages suppressed CM-induced expression of p-NF-κB. (**B**) The protein expression of COX2 was detected by western blot analysis. COX2 expressions in inflammatory H9C2 cells were inhibited by QSG. All data are presented as means ± SEM from independent experiments performed in triplicate. Comparisons were made between CM-treated group and each of the other groups. *P < 0.05, **P < 0.01, ***P < 0.001.

Immunofluorescence results showed that QSG could inhibit the cytoplasm expression of phosphorylated NF-κB (p-NF-κB) in CM-stimulated H9C2 cells (Fig. 4A). Western blot analysis showed that expression of COX2 in CM-stimulated H9C2 cells was reduced when cells were pretreated with QSG (Fig. 4B). Taken together, these results suggested that QSG inhibited CM-stimulated inflammation in H9C2 cells by acting on TNF-α/p-NF-κB pathway and suppressing the expression of COX2.

### Establishment of AMI mice model

**Figure 5:**
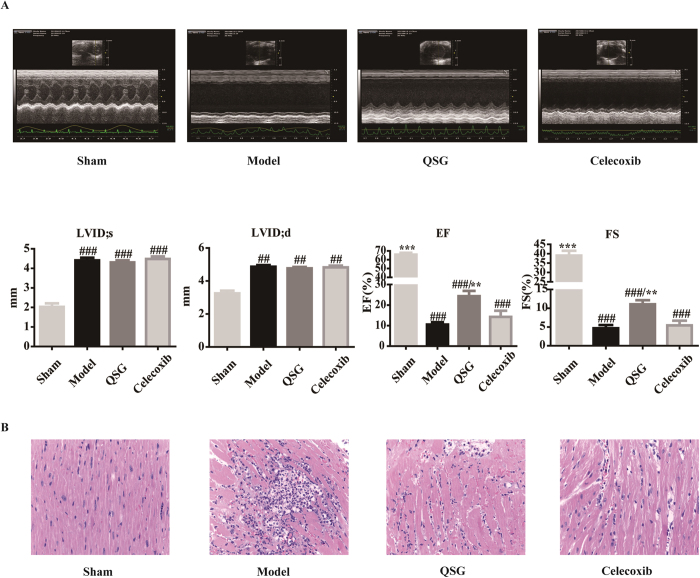
QSG improved cardiac function and suppressed inflammation. (**A**) Cardiac function in each group was detected by echocardiography. QSG improved left ventricular fractional shortening (FS) and left ventricular ejection fraction (EF). All data were presented as means ± SEM from independent experiments performed in triplicate. *P < 0.05, **P < 0.01, ***P < 0.001 vs model group; ^#^*P* < 0.05, ^##^*P* < 0.01, ^###^*P* < 0.001 vs sham group. n = 10 per group. (**B**) QSG preserved cardiomyocyte architecture and inhibited inflammatory cell infiltration.

We established an AMI mice model by ligating left anterior descending coronary artery^13^,^16^. 24 h after surgery, echocardiography showed that LVID;d and LVID;s in the model group increased significantly compared with those in the sham group, suggesting that enlargement of the cardiac chamber occurred at this stage (Fig. 5A). Meanwhile, values of EF and FS in the model group decreased significantly compared with those in the sham group, indicating that cardiac functions were severely impaired in the model group (Fig. 5A). To investigate the effects of QSG on myocardial ischemia, mice in QSG group were treated with QSG for 7 days before undergoing LAD. Values of EF and FS in QSG group were up-regulated compared with those in the model group, suggesting that QSG possessed cardio-protective properties after AMI. Unlike QSG, Celecoxib had no significant effect on cardiac functions (Fig. 5A).

Structures of left ventricular myocardium were well arranged in sham group, as shown by HE-staining of myocardial tissue (Fig. 5B). Large necrotic areas with inflammatory cell infiltration could be observed in the model group. The surrounding cardiomyocytes were arranged in a random pattern. Pretreatment with QSG and Celecoxib rescued hearts from inflammatory infiltration and structural damages (Fig. 5B).

### QSG attenuated cardiac inflammation by regulating arachidonic acid (AA) metabolism pathway

**Figure 6:**
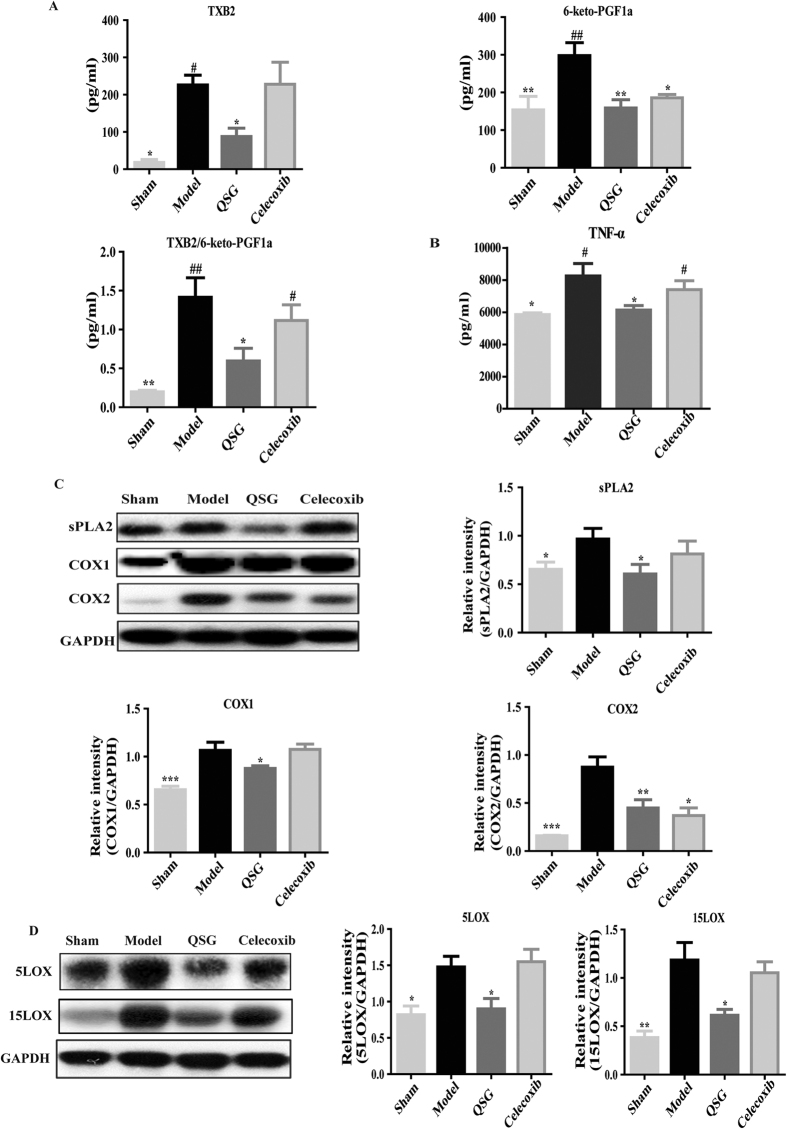
QSG inhibited myocardial inflammation injury though regulating arachidonic acid metabolism. (**A**) The concentrations of TXB2 and 6-keto-PGF1α in plasma in each group are detected through RIA. The plasma level of TXB2 and the ratio of TXB2/6-keto-PGF1α were decreased and 6-keto-PGF1α was increased in QSG group compared with those in model group. (**B**) Elisa showed that QSG reduced the expression of TNF-α in cardiac tissue. (**C**) Western blot showed that QSG reduced the expressions of sPLA2, COX1 and COX2 in cardiac tissue while celecoxib only had effect on COX2. (D) QSG down-regulated the expressions of 5LOX and 15LOX in cardiac tissue while celecoxib had no effect on them. All data were presented as means ± SEM from independent experiments performed in triplicate. *P < 0.05, **P < 0.01, ***P < 0.001 vs model group; ^#^*P* < 0.05, ^##^*P* < 0.01 vs sham group. N = 3 per group.

The effects of QSG on inflammatory pathways were further investigated. Arachidonic acid (AA) metabolism has been shown to be involved in inflammatory process and we examined the effects of QSG on pivotal molecules in this pathway^8^. TXB2 and 6-keto-PGF1α are the metabolite products of AA. In ischemic heart tissues, expression of TXB2 was up-regulated and expression of 6-keto-PGF1α was down-regulated (Fig. 6A). The expression ratio of TXB2 to 6-keto-PGF1α was significantly increased in model group. Pretreatments with QSG reduced expression of TXB2 and up-regulated expression of 6-keto-PGF1α. The expression ratio of TXB2 to 6-keto-PGF1α was regulated towards normal levels (Fig. 6A). Compared with QSG, Celecoxib had the same efficacy on 6-keto-PGF1α, but failed to restore the level of TXB2. Content of TNF-α was upregulated in the model group whereas QSG could effectively inhibit expression of TNF-α (Fig. 6B). PLA2, COX1 and COX2 are the key enzymes in AA metabolism pathway and many drugs, including Celecoxib, are the specific inhibitors of COX2^17^. Western blot analyses showed that QSG inhibited expressions of PLA2, COX1 and COX2 in mice ischemic heart tissue, whereas Celecoxib only suppressed expression of COX2 (Fig. 6C). Furthermore, expressions of 5LOX and 15LOX were also activated in ischemic heart tissues and pretreatment with QSG significantly down-regulated the levels of these two enzymes (Fig. 6D). Celecoxib had no effect on the lipoxygenase pathway including 5LOX and 15LOX (Fig. 6D). Taken together, these results demonstrated that QSG could attenuate inflammation by regulating AA-COXs/LOXs metabolism.

### QSG attenuated cardiac inflammation by inhibiting expressions of JAK2/STAT3

**Figure 7:**
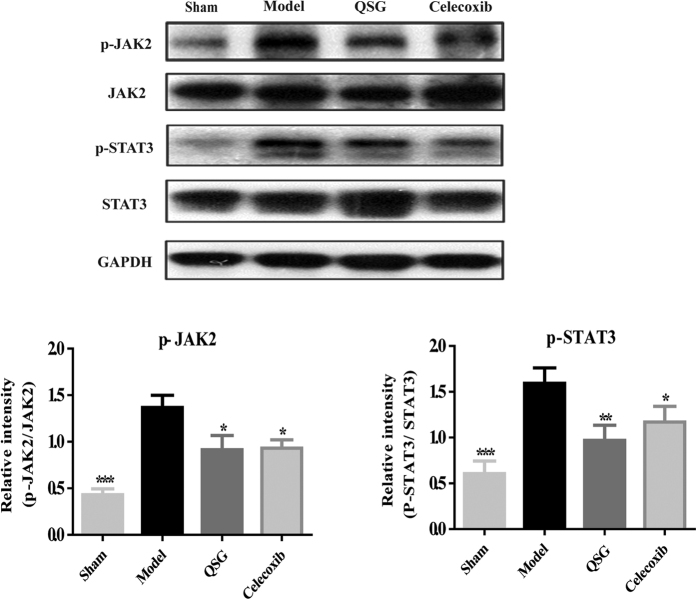
QSG inhibited p-JAK2-STAT3 pathway. QSG down-regulated the ratio of p-JAK2/JAK2 and p-STAT3/STAT3 at the protein levels in heart. *P < 0.05, **P < 0.01, ***P < 0.001 vs model group. N = 3 per group.

Besides AA metabolism, JAK2/STAT3 pathway also plays an important role in inflammatory process. Therefore, we detected both the phosphorylated and unphosphorylated forms of JAK2 and STAT3. Western blot showed that expressions of phosphorylated JAK2 and STAT3 were up-regulated in model group. Pretreatment with QSG significantly down-regulated expressions of activated forms of JAK2 and STAT3, indicating that QSG could also inhibit JAK2/STAT3 pathway, thereby suppressing inflammatory process (Fig. 7).

## Discussion

Our previous study showed that QSG exerted cardio-protective effect and prevented left ventricular remodeling by inhibiting inflammation in AMI model^13^. In this study, we conducted extensive *in vitro* and *in vivo* experiments to further explore the anti-inflammatory mechanisms of QSG in treating AMI. Our main findings are as follows: 1. QSG inhibited release of cytokines from LPS-stimulated RAW 264.7 macrophages. 2. QSG protected H9C2 cardiac cells against CM-induced injury. 3. QSG could improve cardiac functions and alter pathological changes in mice model of AMI. 4. QSG inhibited inflammation by modulating multiple targets in PLA2-COXs/LOXs, TNF-α-NF-κB and IL-6-JAK2-STAT3 signaling pathways.

Inflammation is one of the key mechanisms in promoting coronary heart disease^18^. Arachidonic acid (AA) and its eicosanoid metabolites play a central role in regulating inflammatory signaling pathway^8^. Under extracellular stimuli, AA will be hydrolyzed by the rate-limiting action of PLA2 and serves as a precursor for eicosanoids, which are generated by pathways dependent on COXs, LOXs or cytochrome p450 monooxygenase^19^. COX-1 is constitutively expressed and serves to maintain normal cardiac homeostasis, whereas COX-2 is believed to be induced in various pathophysiologic states and serves as mediator of inflammatory responses which can lead to myocardial damage^13^,^20^,^21^,^22^. COXs convert AA into bioactive prostaglandins, including PGE2 and TXA2^23^. PGE2 is one of the most abundant prostaglandins and involved in all processes leading to inflammation^24^. In LOXs dependent signaling pathway, LOXs catalyze the oxidation of the olefinic linkages of AA to produce HETEs, which are then reduced to HETE derivatives^25^. The 5LOX enzyme catalyzes the conversion of AA into leukotrienes, which play a key role in the pathogenesis of inflammation^26^,^27^,^28^. 15LOX up-regulate expressions of pro-inflammatory cytokines^29^,^30^,^31^. 5LOX and 15LOX have been shown to participate in cardiovascular diseases^29^,^32^. Targeting key enzymes in PLA2-AA signaling pathway to inhibit inflammation is an effective strategy in treating cardiovascular diseases. Celecoxib, the selective COX-2 inhibitor and aspirin, the non-selective COX inhibitor, are among the most widely prescribed drugs. However, these drugs can cause serious side effects, such as perforations, obstructions and gastrointestinal bleedings^33^. Developing new drugs with multiple targets in inflammatory pathways could be an attractive alternative in the management of coronary heart disease.

QSG have been shown to have definitive anti-inflammatory and anti-fibrosis efficacy in treating coronary heart disease^13^,^16^. Our results showed that LPS triggered secretion of proinflammatory cytokines from macrophages, including TNFα and MCP-1. Treatment with QSG inhibited release of these cytokines. Activation of NF-κB plays a central role in inflammation through its ability to induce transcription of proinflammatory genes^34^,^35^. QSG inhibited phosphorylation of NF-κB in macrophages. These results indicated that QSG could alleviate LPS-induced activation of macrophages. We further explored the effects of QSG on H9C2 cardiomyocytes. A CM-stimulated inflammatory H9C2 model was established first. This culture system has been successfully used in the study of neurodegenerative and other diseases^36^,^37^,^38^,^39^. However, it has rarely been used in cardiomyocytes researches. To our knowledge, this CM-stimulated inflammatory H9C2 model was the first one reported by us. Our results demonstrated that treatment of H9C2 cardiac cells with CM induced cytotoxicity, whereas pretreatment of H9C2 cells with QSG protected H9C2 against CM-induced apoptosis and cytotoxicity. Importantly, QSG could remarkably reduce expressions of NF-κB, COX2 and PGE2, suggesting that the anti-inflammatory effect of QSG may be mediated by the regulation of NF-κB and PLA2-COXs pathway.

In addition to *in vitro* studies, we also established a mice model of AMI induced by left anterior descending coronary artery ligation. Treatment with QSG significantly improved compromised cardiac functions and reduced inflammation in mice model. The cardio-protective effect of QSG was more remarkable than that of Celecoxib. Expressions of PLA2, COX1, COX2 and TXB2 in heart tissues were down-regulated and expression of 6-keto-PGF1α was up-regulated by QSG. Furthermore, QSG were also shown to modulate PLA2-LOXs pathway by down-regulating expressions of 5LOX and 15LOX. In contrast, Celecoxib only suppressed expression of COX2 and showed no effect on other molecules in PLA2-COXs/LOXs pathways. Last but not least, QSG inhibited expressions of phosphorylated JAK2 and STAT3 which mediate inflammatory responses^40^,^41^. These *in vivo* results indicated that QSG could act on multiple targets in inflammatory pathways to achieve a synergistic cardio-protective effect.

**Figure 8:**
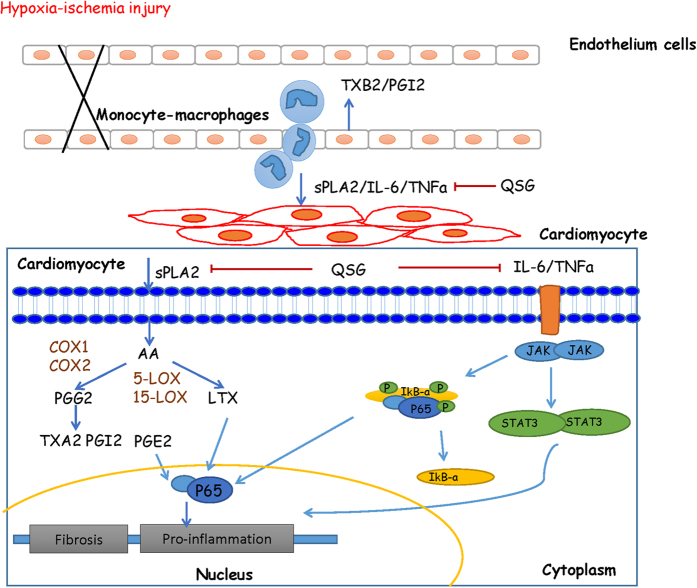
Potential anti-inflammation mechanism of QSG. QSG regulated multiple key molecules, including phospholipases A2 (PLA2), cyclooxygenases (COXs) and lipoxygenases (LOXs), in arachidonic acid metabolism pathway. Moreover, QSG also targeted TNF-α-NFκB and IL-6-JAK2/STAT3 signaling pathways.

In conclusion, this study provides insights into the pharmacological anti-inflammatory mechanism of QSG in treating AMI and inhibiting its progress to HF. The anti-inflammatory effect of QSG might be mediated by suppression of PLA2-COXs/LOXs pathway and JAK2-STAT3 signaling pathway (Fig. 8). This study illustrates that regulating multiple targets in inflammatory pathways could achieve synergistic effect in treating AMI and provides insights into alternative therapeutic approach in the prevention of HF after AMI.

## Methods and Materials

This study was approved by the Animal Care Committee of Beijing University of Chinese Medicine and all animal experiments were performed in accordance with the guidelines on humane use and care of laboratory animals for biomedical research published by National Institutes of Health (No. 85-23, revised 1996).

### Materials

Dulbecco Modified Eagle Mediumas (DMEM) supplemented with 10% Fetal Bovine Serum (FBS), Penicillin (100 U/mL) and Streptomycin (100 μg/mL) was purchased from Corning (New York, USA). LPS was purchased from Sigma Chemical Co. (St. Louis, USA). QSG used in the present study were manufactured by Beijing University of Chinese Medicine (Beijing, China). QSG are composed of 460 g *Radix Astragali Mongolici*, 230 g *Salvia Miltiorrhiza Bunge*, 160 g *Flos Lonicerae*, 160 g *Scrophularia*, 140 g *Radix Aconiti Lateralis Preparata*, and 90 g *Radix Glycyrrhizae*, as described before^13^,^42^. Celecoxib was obtained from Pfizer Pharmaceutical Co., Ltd. (Tianjin, People’s Republic of China). Cell Counting Kit-8 (CCK-8) was purchased from Dojindo molecular technologies, Inc. (Tokyo, Japan). Nitric Oxide (NO) assay kit was purchased from Beyotime Biotechnology (Shanghai, China). Lactate dehydrogenase (LDH) Cytotoxicity Assay Kit was purchased from Applygen technologies, Inc. (Beijing, China). ELISA kits for the detection of TNF-α, monocyte chemotactic protein 1 (MCP-1) and prostaglandin E2 (PGE2) were purchased from Boster Immunoleader (Wuhan, China). Radioimmunoassay (RIA) kits for the detection of thromboxane B2 (TXB2) and 6-keto-prostaglandin F1α (PGF1α) were purchased from Beijing Kangyuan Ruide Biotechnology Co. Ltd.(Beijing, China).

### Culturing of RAW264.7 macrophages

RAW 264.7 macrophages used in this study were obtained from China Infrastructure of Cell Line Resources (Institute of Basic Medical Sciences, Chinese Academy of Medical Sciences). RAW 264.7 were incubated with DMEM supplemented with 10% Fetal Bovine Serum (FBS), Penicillin (100 U/mL) and Streptomycin (100 μg/mL) at 37 °C in a humidified atmosphere of 5% CO_2_ and 95% air. To evaluate the effects of QSG on LPS-activated RAW264.7 cells were exposed to LPS (1 μg/mL) for 24 h with or without QSG (added to media 2 h before treatment with LPS). The concentrations of QSG in culture media in different cell groups were 0, 400, 600, 800 and 1000 μg/mL, respectively. Celecoxib was added to one cell group 2 h prior to treatment with LPS and this group was positive control group. Cell supernatants were collected and stored at −20 °C for further analysis. For cell viability test, approximately 1 × 10^5^ cells/well of RAW 264.7 cells were seeded into 96-well plates. For western blot analysis, 2 × 10^6^cells/well RAW 264.7 cells were seeded in 6-well plates.

### Culturing of H9C2 cardiac cells

Rat myocardial H9C2 cells purchased from China Infrastructure of Cell Line Resources (Institute of Basic Medical Sciences, Chinese Academy of Medical Sciences) were cultured in DMEM supplemented with 10% Fetal Bovine Serum (FBS), Penicillin (100 U/mL) and Streptomycin (100 μg/mL) at 37 °C under a humidified atmosphere of 5% CO_2_ and 95% air. In all experiments, cells were allowed to synchronize for 4 h before any treatment. To induce inflammation, H9C2 cells were incubated with different concentrations of LPS (10, 5 and 1 μg/mL) for 24 h. For measuring nitrite concentrations, LDH and cell viability, H9C2 cells were seeded 10^4^ cells/well in 96-well plates. Supernatant liquid from RAW264.7 cells pretreated with 1 μg/mL LPS for 24 h was collected as macrophage-conditioned media (CM). As LPS treatment alone was not sufficient to induce inflammatory reactions in H9C2 cells in our study, we established a novel CM-induced cardiac inflammatory cell model. Specifically, CM collected from LPS-stimulated RAW264.7 was administered to H9C2 cells for 24 h. Besides, CM was diluted to one third (CM/3) and one sixth (CM/6) of its original concentration by DMEM and applied for induction of inflammation in H9C2 cells. To investigate the effects of QSG on inflammation in CM-stimulated H9C2 cells, H9C2 cells were pretreated with QSG (400,600,800,1000 μg/mL) for 6 h and then cells were incubated with CM for 24 h. Cell supernatants were collected and stored at −20 °C for further analysis. For ELISA essay and western blotting analysis, 3 × 10^5^/ well H9C2 cells were seeded in 6-well plates. For immunofluorescent assay, 2 × 10^4^ H9C2 cells were seeded in confocal laser small dish.

### Measurement of cell viability

Cell Counting Kit-8, a commercially available cell viability assay, was employed to evaluate the cytotoxic effect. Approximately 1 × 10^5^ of RAW 264.7 cells or 1 × 10^4^ of H9C2 cells was seeded into 96-well plates and then incubated with various concentrations of LPS or QSG for 24 h at 37 °C in a 5% CO_2_ incubator. Cells not treated with LPS or QSG served as a control well in the experiment. Afterwards, 10 μL of CCK-8 solution was added to each well for 2 hour, and then absorbance was determined at 450 nm by a DTX-880 multimode microplate reader (Beckman Coulter, Fullerton, CA, USA). The percentage of cell viability was calculated by the following formula: cell viability (%) = (mean absorbency in test wells)/(mean absorbency in control wells) × 100%. All experiments were performed in triplicate.

### Measurement of NO, LDH and inflammatory cytokines

Cell supernatants were collected from LPS-stimulated RAW 264.7 (with or without QSG pretreatment) and CM-activated H9C2 cells (with or without QSG pretreatment) for the detection of NO and LDH released from cultured cells. NO production was determined by NO assay kit based on Griess method and LDH production was determined by LDH Cytotoxicity Assay Kit. The concentrations of TNF- α, MCP-1 and PGE2 in the supernatants were determined by ELISA kits, according to the manufacturer’s instructions. Standards at a series of concentrations were run in parallel with the samples. The concentrations in the samples were calculated in reference to the corresponding standard curves and were expressed as ng/mL.

### Immunofluorescence assay

H9C2 cells were fixed with 4% paraformaldehyde for 20 min in confocal laser small dish followed by permeabilization (0.5% Triton X100 in PBS) and blocking (5% sheep serum in PBS) for 30 min at room temperature. Cells were then incubated overnight with primary antibody (1:250) at 4 °C and with a secondary antibody (FITC, 1:500) for 1 h at room temperature. The cells were further stained with DAPI (5 μg/mL in PBS) at 37 °Cfor 20 min. Finally, the cells were sealed on cover slips and images were acquired using OLYMPUS IX73 fluorescence microscope (Tokyo, Japan) with excitation/emission wavelengths of 590 nm/617 nm for Alexa Fluor-594 and 360 nm/450 nm for DAPI.

### Establishment of AMI model

A total of 60 male C57/BL6 mice aged between 10–12 weeks and weighed 25–28 g in SPF grade purchased from Beijing Sibei Fu Biotechnology Co., Ltd. (Beijing, China) were used in this study. Left anterior descending coronary artery (LAD) of mice was ligated for the establishment of animal model of AMI as previously described^43^. Briefly, C57/BL6 mice were put under general anesthesia with 0.5% pentobarbital sodium and ventilated by a rodent respirator. After a left thoracotomy between the third and fourth intercostal space, the heart was exposed and LAD was ligated with a 7–0 silk suture 4 mm below the left atrium in AMI group, whereas in the sham operated group, the needle was passed around the artery without ligation. The thorax was then closed layer by layer with a continuous 5–0 prolene suture and the mice were allowed to recover.

Mice were randomly divided into four groups: sham operated group, model group, QSG group and Celecoxib positive control group. The dosage of QSG administered to mice per day was 3.33 g/kg and the dosage of Celecoxib was 33.3 mg/kg, as described before^13^,^16^. QSG and Celecoxib solutions were orally administrated to mice 7 d prior to LAD ligation. After LAD ligation, mice were fed with normal diet for twenty four hours before being sacrificed.

### Echocardiography

Echocardiography was performed in mice anesthetized with 1.5–2% isoflurane (AbraxisBioScience, Richmond Hill, Ontario, Canada) using a Vevo 2100 (VisualSonics Inc, Toronto, Ontario, Canada) with a 21 MHz probe central frequency scan head. The following parameters were measured from M-mode images taken from the parasternal short-axis view at papillary muscle level: left ventricular internal dimension-diastole (LVID;d), left ventricular internal dimension-systole (LVID;s), left ventricular fractional shortening (FS) and left ventricular ejection fraction (EF).

### Histological examination

Hematoxylin-eosin (HE) staining was performed to visualize cardiomyocyte architecture. The heart tissue in the left ventricle (LV) of sacrificed rats (approximately 2 mm in thickness) were removed, fixed in 4% paraformaldehyde solution for more than 48 h, and further prepared for paraffin sectioning. Serial sections (5 μm) were cut and stained with hematoxylin–eosin. The sections were analyzed under light microscope. Images were visualized under an optical microscope at 400x magnification.

### Determination of TXB2 and 6-keto-PGF1α by RIA

The plasma was homogenized in saline containing enzyme inhibitor (0.3 M EDTA-Na 10 μL, 0.34 M 8-hydroxyquinoline 10 μL, 0.32 M dimercaptopropanol 5 μL) (1 mL blood) on ice. The homogenate was centrifuged at 3000 g for 10 min. The supernatants were used for the determination of TXB2 and 6-keto-PGF1α using a RIA kit following the instructions of the company.

### Western blot analysis

Cardiac tissues were lysed using RIPA buffer (50 mM Tris–HCl Ph7.4, 150 mM NaCl, 1% NP-40 and 0.1% SDS) containing a protease inhibitor cocktail (Sigma, St. louis, MO, USA). Equal amounts of protein were subjected to SDS-PAGE and transferred onto PVDF membranes. Standard western blot analysis was conducted using Transforming growth factor beta (TGF-b, 1:1000 dilution; Cell Signaling Technology, Boston, Massachusetts, USA). Glyceraldehyde 3-phosphate dehydrogenase antibody (GAPDH, 1: 1000 dilution; Kangchen, Shanghai, China) was used as a loading control. After incubation with the appropriate secondary antibodies, signals were visualized using the ECL Plus Western blotting detection reagents (Bio-Rad) for 1 min at room temperature. The bands in the membrane were visualized and densitometric analysis of band intensity was performed using Imagelab software (Bio-Rad, Hercules, CA, USA). Then the same procedure was taken to detect the others proteins using their antibodies respectively, such as Secretory phospholipase A2 (sPLA2, 1:1000 dilution;Abcam, Cambridge, UK), Bax (Rabbit monoclonal to anti-Bax: Abcam, USA. Ab32503), Bcl-2(Rabbit polyclonal to anti-Bcl-2: Abcam, USA. Ab7973), Caspase3(Rabbit monoclonal to anti-Caspase3: CST, GERMAN, 9665), p-NF-κB (anti- NFκB p65: Abcam, USA, Ab32536), COX1(Rabbit monoclonal to anti-COX1: Abcam, USA. Ab109025), COX2 (Rabbit monoclonal to anti-COX2: Abcam, USA. Ab15191), 5 Lipoxygenase (Rabbit monoclonal to anti-5 Lipoxygenase: Abcam, USA. Ab169755), 15 Lipoxygenase (Mouse monoclonal to anti-15 Lipoxygenase 1: Abcam, USA. Ab119774), P-JAK2 (Rabbit monoclonal to anti-P-JAK2: CST, German, 3776), JAK2 (Rabbit monoclonal to anti-JAK2: CST, German, 3230), P-STAT3 (Mouse monoclonal to anti- P-STAT3: CST, German, 4113) and STAT3 (Rabbit monoclonal to anti-STAT3: CST, German, 4904). For *in vitro* study, cells were digested by trypsin and the collected cells were prepared with cell lysis and proteins were extracted according to the manufacture’s instruction. Protein contents were measured with BCA Bradford protein assay (PPLYGEN, China).

### Statistical analysis

All data were expressed as mean ± SE Mean (SEM). Statistical analyses were performed using one-way analysis of variance (SPSS 17.0 statistical software or GraphPad Prism 5). Tukey’s and Dunnett tests were applied for multiple comparisons between groups. *P* < 0.05 was considered as statistically significant.

## Additional Information

**How to cite this article**: Li, C. *et al*. Qishen granules inhibit myocardial inflammation injury through regulating arachidonic acid metabolism. *Sci. Rep.* **6**, 36949; doi: 10.1038/srep36949 (2016).

**Publisher’s note:** Springer Nature remains neutral with regard to jurisdictional claims in published maps and institutional affiliations.

## Supplementary Material

Supplementary Information
