# Supplementary material for: Qishen granules inhibit myocardial inflammation injury through regulating arachidonic acid metabolism
Source: Sci Rep. 2016 Nov 11;6:36949. doi: 10.1038/srep36949 (PMC5105076; doi:10.1038/srep36949)
Supplement: Supplementary Information [file srep36949-s1.pdf]

Qishen granules inhibit myocardial inflammation injure through regulating arachidonic acid metabolism

Chun Li [1, †], Jing Wang [1, †], Qiyan Wang [2], Yi Zhang[3], Na Zhang[1] , Linghui Lu [2], Yan Wu [4], Qian Zhang [2], Wei Wang [2], Yong Wang [2,*], Pengfei Tu [1,*]

[1] Modern Research Center for Traditional Chinese Medicine, Beijing University of Chinese Medicine, Beijing 100029, China

[2] Basic Medical College, Beijing University of Chinese Medicine, Beijing 100029, China

[3] School of Chinese Materia Medica, Beijing University of Chinese Medicine, Beijing 100102, China

[4] Center of Scientific Experiment, Beijing University of Chinese Medicine, Beijing 100029, China

SUPPLEMENTARY DATA 1: HPLC-PDA chromatogram of QSG

HPLC-grade acetonitrile is product of Fisher Scientific (FairLawn, NJ, USA). Formic acid is product of Sigma Aldrich (St. Louis, MO, USA). Ultrapure water was prepared in our own laboratory by Milli-Q plus System (Millipore, Bedford, MA, USA). Analytical-grade solvents used for sample preparation are products of Beijing Chemical Factory (Beijing, China).

Sample preparation for analysis

QSG was weighed accurately (0.04 g) and placed into a 1.5 mL centrifuge tube containing 1.0 mL 50% aqueous methanol for 5 minutes in a vortex. Following centrifugation at 12000 rpm for 10 minutes in a centrifuge (Eppendorf, Melbourne, Australia). A supernatant (200 μL) was 5-fold diluted with 50% aqueous methanol and centrifuged at 12000 rpm for 10 minutes.

HPLC-IT-TOF-MS^n conditions for chemical profile

The HPLC analysis was carried out on a Shimadzu HPLC (two LC-20AD$_{XR}$ solvent delivery units，a SIL-20AC$_{XR}$ auto-sampler, a CTO-20AC column oven, a SPD-M20A PDA detector, a DGU-20A$_{3R}$ degasser, and a CBM-20A controller). The chromatographic separation was

performed on a Shiseido C_{18} column (150 × 2.1mm, 2.7 µm) at 35 ºC. 0.02% aqueous formic acid
B
(A) and acetonitrile containing 0.02% formic acid (B) –were used as the mobile phase for analysis. The flow rate was set at 0.4 mL/min. The elution condition was applied with a gradient program as follows: 0–20 min, 2-18% B; 20–30 min, 18–30% B; 30–35 min, 30–45% B; 35–40 min, 45–65% B; 40–55 min, 65–95% B; 55–60 min, 95% B. 10 µL were injected into HPLC system for analysis. The typical chromatograms are shown in Fig. 1

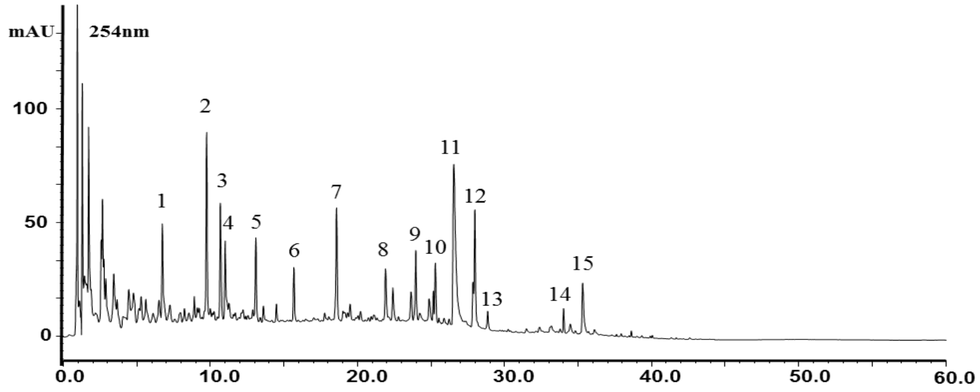

Fig 1. HPLC-PDA chromatogram of QSG at 254nm

1. Chlorogenic acid; 2. Cryptochlorogenin Acid; 3. Neochlorogenic acid; 4. Secologanic acid; 5. Sweroside; 6. Secoxyloganin; 7. Liquiritin; 8. Isochlorogenic acid A; 9. Isochlorogenic acid C; 10. Ononin; 11. Salvianolic acid B; 12. Calycosin; 13. Harpagoside; 14. Formononetin; 15. Glycyrrhizic Acid

SUPPLEMENTARY DATA 2: The primary data of echocardiographic assessment of left ventricular function

EF

Sham	Model	QSG	Celecoxib
66.4836	14.1837	29.4668	17.3568
58.6966	7.8021	16.3705	14.3530
55.6926	2.3079	10.0835	7.5542
66.9929	14.3269	32.6706	26.3529
64.9829	13.0286	25.2978	15.0863
60.4057	12.8983	18.1503	14.5160
58.4772	7.7240	14.0543	13.8699
77.6966	23.5264	53.0403	28.6639
57.2960	2.3312	13.6689	9.1352
73.9967	23.2912	45.5361	27.5804

FS

Sham	Model	QSG	Celecoxib
35.4783	5.9048	11.1878	7.7273
31.5951	3.5088	7.7626	6.1005
28.6119	1.0138	5.2361	3.2630
36.4985	6.0935	15.5251	7.8341
35.3846	5.7867	10.4722	6.5259
35.0000	5.1441	8.8813	6.3636
29.7222	3.4386	5.7970	5.3812
42.5739	8.7050	22.3757	13.4529
29.5652	1.0345	5.5939	3.9170
42.0382	6.4302	20.9444	8.2010

LVID;d

Sham	Model	QSG	Celecoxib
3.7620	5.0869	5.1678	4.7504
2.7857	4.1339	4.0514	4.4332
2.5081	3.2657	3.1667	3.7521
3.9475	5.5360	5.7018	4.9424
3.1948	4.9562	4.7981	4.6227
3.1667	4.5672	4.5500	4.4531
2.6604	4.0173	3.7775	4.1900
4.9972	6.8333	6.4971	6.3825
2.6088	3.3588	3.4979	3.9665
4.3639	5.9883	5.9617	5.7014

LVID;s

Sham	Model	QSG	Celecoxib
2.4273	4.7866	4.5076	4.3782
1.9056	3.9888	3.8247	4.1627
1.7905	3.2326	2.9831	3.6296
2.5067	5.1987	4.5897	4.5370
2.0644	4.6694	4.2957	4.3210
2.0583	4.3322	4.1968	4.1698
1.8697	3.8792	3.5797	3.9645
2.8697	6.2384	5.0433	5.5238
1.8375	3.3240	3.1873	3.8111
2.5294	5.6032	5.0361	5.2608
